# Successful management of a patient with preoperative respiratory failure due to a solid giant retroperitoneal tumor: a case report

**DOI:** 10.1186/s40981-022-00575-8

**Published:** 2022-10-17

**Authors:** Hirotaka Kinoshita, Eiji Hashiba, Satoshi Uchida, Kazuyoshi Hirota

**Affiliations:** 1 Department of Anesthesiology, Hirosaki University Graduate School of Medicine, 5 Zaifu-cho, Hirosaki, 036-8562 Japan; 2 Division of Intensive Care, Hirosaki University Hospital, 53 Hon-cho, Hirosaki, 036-8563 Japan

**Keywords:** Giant tumor, Respiratory failure, Hemodynamic system, Nasal high-flow therapy, ROX index

## Abstract

**Background:**

We report the successful preoperative management of respiratory failure in a patient with a solid giant retroperitoneal tumor with a hemodynamic monitoring system and nasal high-flow therapy (NHFT).

**Case presentation:**

Twenty days before his scheduled resection of a giant retroperitoneal liposarcoma, a 64-year-old man presented with dyspnea. After admission to our intensive care unit, he received NHFT and hemodynamic therapy using a LiDCOrapid V3™ monitor (Masimo Japan, Tokyo). NHFT and intense diuresis improved his respiratory condition. The tumor resection was performed on the 5th day. He was discharged to the general ward with an oxygen nasal cannula on the second postoperative day. Although preoperative transthoracic echography showed mild aortic regurgitation and moderate mitral regurgitation, the degree of regurgitation had become trivial about 1-month post-surgery.

**Conclusions:**

A cause of preoperative respiratory failure associated with a giant retroperitoneal tumor might be not only diaphragmatic compression but also heart failure and excess fluid volume.

## Background

The perioperative management of patients with a giant retroperitoneal tumor or an ovarian tumor requires strict attention to circulatory and respiratory dynamic changes and the potential development of deep vein thrombosis and pulmonary thrombosis. Perioperative respiratory failure due to these giant tumors is associated with diaphragmatic compression, re-expansion pulmonary edema, and thinning of respiratory muscles [1]. If the tumor contents are liquid, preoperative drainage of liquid component could reduce these respiratory complications [2, 3]; it is not possible to do this if tumor component is solid. Herein, we report the successful management of a patient who presented with respiratory failure before scheduled surgery, with the use of a hemodynamic monitoring system to control the accumulation of body fluid and nasal high-flow therapy (NHFT).

## Case presentation

We have obtained a written informed consent from the patient and his family for the publication of this case report.

**Fig. 1:**
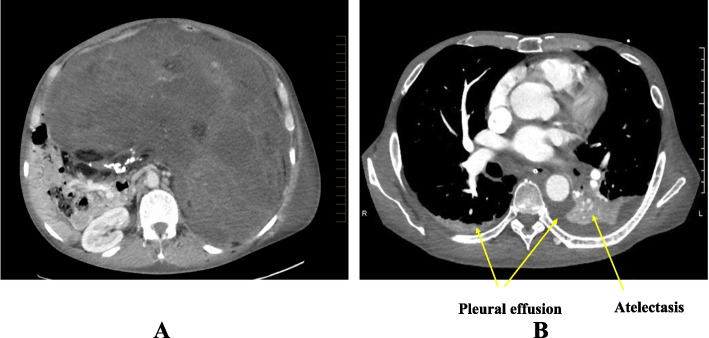
The patient’s CT images on admission to the intensive care unit (ICU). A giant retroperitoneal tumor occupied his abdominal and pelvic region, and severe compression of the inferior vena cava (**A**), bilateral pleural effusion, and atelectasis (**B**) were observed

A 64-year-old man (height 175 cm, weight 85 kg) with a giant retroperitoneal tumor was scheduled for tumor resection and left nephrectomy. The tumor component was diagnosed as liposarcoma based on the pathological result of the computed tomography (CT)-guided biopsy before the surgery. He presented with dyspnea 20 days before the scheduled surgery and consulted his attending urologist. Upon admission to the general ward, he experienced respiratory failure and underwent oxygen therapy with a reservoir mask (PaO2/F_I_O2 ratio: 72). His lower legs were markedly edematous. Further CT imaging demonstrated the giant retroperitoneal tumor occupying his abdominal and pelvic region (Fig. 1A), severe compression of the inferior vena cava, bilateral pleural effusion, and atelectasis (Fig. 1B). Transthoracic echography showed normal left ventricular ejection fraction (> 50%) and diastolic function, no regional wall motion abnormality, mild aortic regurgitation, and moderate mitral regurgitation. The patient’s N-terminal pro-brain natriuretic peptide (NT-pro BNP) value was 1232 pg/mL. We thus diagnosed the cause of his respiratory failure as not only diaphragmatic compression due to the giant retroperitoneal tumor but also heart failure and excessive volume. He refused preoperative intubation and ventilator management.

**Fig. 2:**
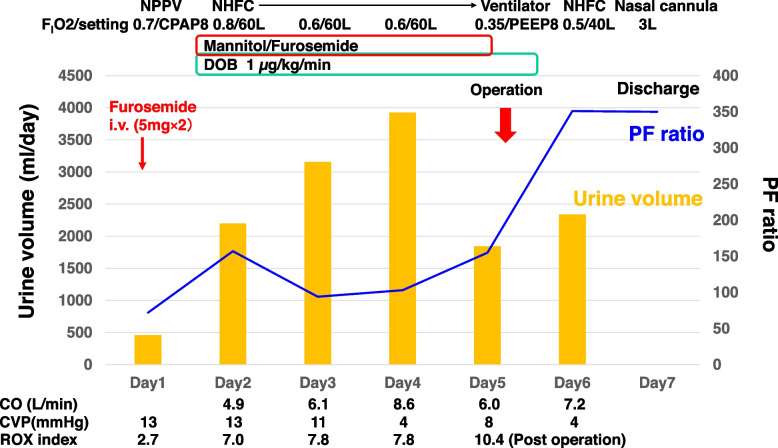
The patient’s progress chart in the ICU. NPPV, noninvasive-positive pressure ventilation; NHFC, nasal high-flow cannula; CPAP, continuous positive airway pressure; PEEP, positive end-expiratory pressure; DOB, dobutamine; CO, cardiac output; CVP, central venous pressure

After being admitted to our intensive care unit (ICU), the patient received noninvasive positive pressure ventilation (NPPV) and hemodynamic therapy with reference to cardiac output and cardiac index provided by LiDCOrapid V3™ (Masimo Japan, Tokyo) monitor. In addition, we inserted central venous pressure catheter to his right internal jugular vein. On the patient’s 2nd day in the ICU, the NPPV changed to NHFT based on the patient’s desire. Due to the lack of efficacy of a single intravenous furosemide infusion, his diuresis was managed with continuous intravenous infusions of dobutamine, mannitol, and furosemide (Fig. 2). The patient’s body weight had decreased from 85 to 78 kg until the operative day; the value of NT-pro BNP decreased to 637 pg/mL immediately before surgery. His ROX index (i.e., the ratio of oxygen saturation as measured by pulse oximetry/F_I_O2 to the respiratory rate [4]) remained over 7.0 after the provision of a NHFT and the decrease in the accumulated body fluid.

On the 5th day after the patient’s admission to the ICU, the retroperitoneal tumor was resected. The tumor specimen was liposarcoma, weighing 17.4 kg. The amount of intraoperative bleeding was 3300 g. From before to after the tumor resection, the cardiac output changed from 6.9 to 10.3 L/min, and the stroke volume variation (SVV) changed from 19.7 to 3.7%. On the day of the tumor resection, the patient was ventilated with 8 cmH_2_O of positive end-expiratory pressure (PEEP) in order to avoid re-expansion pulmonary edema, and he was extubated and managed with NHFT the day after the surgery. On the 2nd postoperative day, the patient was discharged to the general ward in stable respiratory condition with an oxygen nasal cannula. About 1 month after the surgery, transthoracic echocardiography showed that the degree of aortic and mitral regurgitation had become trivial.

## Discussion

Giant retroperitoneal liposarcomas are solid and rare intra-abdominal malignant tumors. They can be asymptomatic as they grow, until they are large enough to compress the surrounding organ [5]. The present patient developed severe respiratory failure before the surgery. Previous case reports suggested that preoperative respiratory failure with a giant intra-abdominal tumor has a high risk of respiratory prognosis [6]. Kanda et al. described a case of respiratory failure with thinning of respiratory muscle due to a giant (43.7 kg) ovarian tumor. The patient could not expectorate sputum by herself and eventually died due to postoperative aspiration pneumonia. However, the cause of an instance of respiratory failure in a patient with a giant tumor might be not only diaphragm compression but also heart failure and excess fluid volume. Giant tumors lead to increase in systemic vascular resistance due to mechanical compression of intra-abdominal and intrathoracic vasculature and compensatory vasoconstriction [7]. The increasing afterload associated with giant tumors plus undernutrition could further exacerbate respiratory compromise due to diaphragmatic compression. Indeed, our patient’s case suggested that his preoperative respiratory failure and giant tumor were associated with heart failure and excessive fluid volume, based on the NT-pro BNP and echocardiography results. An increasing afterload with the giant tumor may have exacerbated valve regurgitation, resulting in congestive heart failure. Supporting this possibility, the patient’s postoperative transthoracic echocardiography demonstrated that the degree of aortic and mitral regurgitation had become trivial at 1-month post-surgery. However, surgical resection is preferred in Meigs syndrome with similar symptoms such as respiratory failure and pleural effusion because diuretics are ineffective [8].

It is also known that careful preoperative drainage of the liquid-type contents (such as the contents of ovarian tumors) can reduce the likelihood of respiratory complications after the surgery [2, 3, 9]. In the present patient, due to the tumor’s solidity, it was impossible to reduce the mass effects of the giant retroperitoneal liposarcoma immediately by draining the tumor contents, and the emergency surgery could not be conducted because of the patient’s unstable physical condition and the highly difficult operation which needed special surgical team with urological and gastroenterological surgeons. We applied NPPV, NHFT, and intense diuresis with a continuous infusion of the furosemide and mannitol while avoiding the decrease in cardiac output monitored by the LiDCOrapid V3™ hemodynamic monitoring system. The patient’s pulmonary atelectasis at the left lower lobe and the marked edema of his lower body gradually improved before the surgery.

The efficacy of a diuretic drug led to a decrease in the patient’s preoperative NT-pro BNP value from 1232 to 637 pg/ml. However, even with the preoperative fluid managements, the cardiac output increased from 6.9 to 10.3 L/min, and the SVV decreased from 19.7 to 3.7% after the tumor resection during the general anesthesia. If the patient underwent tumor resection without any preoperative fluid managements, he might have more congestive lung conditions due to postoperative over fluid. The preoperative control of excess fluid will be advantageous for the patients with a giant intra-abdominal tumor. In addition to the above-described treatment, the patient was ventilated and managed with relatively high PEEP on the day of surgery to prevent re-expansion pulmonary edema.

In this case, we were unable to intubate and ventilate the patient’s lung preoperatively due to the patient’s refusal. Another study showed that NPPV received the worst subjective ratings, although it led to the best improvement in oxygenation with powerful assist effects [10]. The present patient also complained of aversion to NPPV, and the change to NHFT increased his rating of his discomfort and dyspnea. Following these treatments, the patient’s ROX index was improved from 2.7 to 7.0. A prospective observational cohort study reported that a ROX index ≥ 4.88 measured after 12 h of NHFT was significantly associated with a lower risk of the need for mechanical ventilation [4]. The ROX index has good discriminating power for the prediction of NHFT failure in COVID-19 patients with acute hypoxemic respiratory failure [11]. The present patient’s ROX index remained > 7.0 with the administration of NHFC.

In conclusion, we were able to successfully manage a patient with respiratory failure associated with a giant retroperitoneal liposarcoma by using a hemodynamic system and NHFT. This case highlights the possibility that the cause of preoperative respiratory failure associated with a giant retroperitoneal tumor might be not only diaphragmatic compression but also heart failure and excess fluid volume.

## Data Availability

Please contact the corresponding author for data requests.

## Abbreviations

CT: Computed tomography
ICU: Intensive care unit
NHFT: Nasal high-flow therapy
NPPV: Noninvasive positive pressure ventilation
NT-pro BNP: N-terminal pro-brain natriuretic peptide
PEEP: Positive end-expiratory pressure
SVV: Stroke volume variation
